# A diagnosis model for brain atrophy using deep learning and MRI of type 2 diabetes mellitus

**DOI:** 10.3389/fnins.2023.1291753

**Published:** 2023-10-30

**Authors:** Saba Raoof Syed, Saleem Durai M. A.

**Affiliations:** Vellore Institute of Technology, School of Computer Science and Engineering, Vellore, Tamilnadu, India

**Keywords:** brain atrophy, T2DM, MRI, deep learning, TRAU-Net, MLAST, segmentation, classification

## Abstract

**Objective:**

Type 2 Diabetes Mellitus (T2DM) is linked to cognitive deterioration and anatomical brain abnormalities like cerebral brain atrophy and cerebral diseases. We aim to develop an automatic deep learning-based brain atrophy diagnosis model to detect, segment, classify, and predict the survival rate.

**Methods:**

Two hundred thirty-five MRI images affected with brain atrophy due to prolonged T2DM were acquired. The dataset was divided into training and testing (80:20%; 188, 47, respectively). Pre-processing is done through a novel convolutional median filter, followed by segmentation of atrophy regions, i.e., the brain shrinkage, white and gray matter is done through the proposed TRAU-Net model (Transfer Residual Attention U-Net), classification with the proposed Multinomial Logistic regression with Attention Swin Transformer (MLAST), and prediction of chronological age is determined through Multivariate CoX Regression model (MCR). The classification of Brain Atrophy (BA) types is determined based on the features extracted from the segmented region. Performance measures like confusion matrix, specificity, sensitivity, accuracy, F1-score, and ROC-AUC curve are used to measure classification model performance, whereas, for the segmentation model, pixel accuracy and dice similarity coefficient are applied.

**Results:**

The pixel accuracy and dice coefficient for segmentation were 98.25 and 96.41, respectively. Brain atrophy multi-class classification achieved overall training accuracy is 0.9632 ± 1.325, 0.9677 ± 1.912, 0.9682 ± 1.715, and 0.9521 ± 1.877 for FA, PA, R-MTA, and L-MTA, respectively. The overall AUC-ROC curve for the classification model is 0.9856. The testing and validation accuracy obtained for the proposed model are 0.9379 and 0.9694, respectively. The prediction model's performance is measured using correlation coefficient (*r*), coefficient determination *r*^2^, and Mean Square Error (MSE) and recorded 0.951, 0.904, and 0.5172, respectively.

**Conclusion:**

The brain atrophy diagnosis model consists of sub-models to detect, segment, and classify the atrophy regions using novel deep learning and multivariate mathematical models. The proposed model has outperformed the existing models regarding multi-classification and segmentation; therefore, the automated diagnosis model can be deployed in healthcare centers to assist physicians.

## 1. Introduction

Type 2 Diabetes Mellitus (T2DM) is a chronological disorder caused by high glucose levels in the blood for a significant period. T2DM is usually known as hyperglycemia, an acute condition due to reduced insulin synthesis and resistance. Around 90% of Diabetic Mellitus cases are of T2DM, leading to various severe disorders and complications affecting multiple organs if untreated and uncared for a prolonged period. T2DM complications are classified into two types based on the blood vessels affected: microvascular (small blood vessels) and macrovascular (large blood vessels). Generally, type 2 diabetes leads to various health problems, including diabetic retinopathy, neuropathy, nephropathy, vascular diseases, stroke, and heart disease. It also leads to rapid cognitive deterioration, Alzheimer's, dementia, and Cerebral Small Vascular Diseases (CSVD) like brain atrophy (BA), lacunar infracts, and White Matter Hyperintensities (WMH) (Forbes and Cooper, 2013). According to the research done on Type 2 Diabetes Mellitus (T2DM), it appears that it can have an impact on cognitive abilities such as memory, processing speed, and executive function (Kodl and Seaquist, 2008; Cherbuin and Walsh, 2019). The exact mechanism by which T2DM affects cognitive function is not yet fully understood, but several factors have been associated with it, including hyperglycemia, vascular disorders, hypoglycemia, and insulin resistance. These factors contribute to an increased risk of cognitive impairment. Additionally, researchers, some evidence suggests that T2DM may be linked to the development of Alzheimer's disease and CSVD (Kawamura et al., 2012).

Several research studies have revealed that the human brain's Magnetic Resonance Imaging (MRI) can be utilized to identify structural transformations that may be correlated with T2DM. Brain volumetry is a prevalent technique employed to measure the extent of brain atrophy, which has consistently demonstrated that T2DM is linked with a reduction in the average total brain volume. According to research, individuals with type 2 diabetes mellitus (T2DM) experience a mean total brain volume reduction of 0.2–0.6 standard deviation units. This reduction is equivalent to 3–5 years of normal aging, as studies conducted (Van Harten et al., 2006; Cheng et al., 2012; Zhang T. et al., 2022). An increase in the size of the fluid-filled spaces in the brain, known as ventricular enlargement, has been seen in people with type 2 diabetes. This suggests that the subcortical areas surrounding the ventricles, which are important for movement and coordination, may be particularly vulnerable to the effects of T2DM. However, we still do not understand how much brain atrophy is caused by T2DM, which brain structures are most affected, and when these changes occur during old age (De Bresser et al., 2010).

People with T2DM have more brain lesions and white matter hyperintensities than people without T2DM, likely due to the increased vascular damage that occurs in T2DM. The brain structural differences associated with T2DM may also be related to the duration of the disease and blood glucose levels. Studies have also found that T2DM is associated with reduced volume in specific brain regions, such as various cortical regions, basal ganglia, and hippocampus, even in cognitively healthy people (De Bresser et al., 2010; Kooistra et al., 2013).

Furthermore, T2DM has been linked to a higher incidence of brain lesions and greater white matter hyperintensities, most likely due to increased vascular pathology. These differences in brain structure could also be linked to the duration of T2DM and blood glucose levels. Studies that have focused on specific brain regions have discovered that there are negative links between T2DM and the volume of sub-regions, including the hippocampus, basal ganglia, and numerous cortical regions among cognitively healthy individuals (Manschot et al., 2006; Tiehuis et al., 2008; Bruehl et al., 2011; Espeland et al., 2013).

The connection between Type 2 Diabetes Mellitus and brain shrinkage was investigated in a meta-analysis study (Zhang T. et al., 2022). Individuals with T2DM had significantly smaller total brain volume, gray matter volume, white matter volume, and hippocampal volume (~1–4%) (Zhang T. et al., 2022). This paper surveys machine learning techniques applied to structural MRI data to obtain clinical classifiers for various diseases and disorders (Mateos-Pérez et al., 2018). This paper reviews the progress in quantifying the common cerebral small vessel disease (CSVD) neuroimaging features. It explores the clinical consequences of these features and the possibilities of using them as endpoints in clinical trials (Zhao et al., 2021). The research paper (Ghose et al., 2022a) proposes a CNN model for diagnosing COVID-19 from chest X-rays. The model achieved remarkable accuracy, sensitivity, specificity, precision, and F1-score rates, with 98.5, 99.2, 98.9, 99.2, and 98.3%, respectively. It also demonstrated a 99.60% accuracy in distinguishing COVID-19 from pneumonia patients. This model can potentially revolutionize the medical industry's approach to COVID-19 diagnosis, leading to more efficient and effective patient care. Another study (Ghose et al., 2022b) proposes a transfer learning-based approach to detecting COVID-19 infection status from chest radiographs and computed tomography (CT) scans with an accuracy of 99.59 and 99.95%, respectively.

The study in Callisaya et al. (2019) explored the connection between Type 2 Diabetes and brain atrophy and its impact on cognition. The findings revealed that T2DM is linked to increased cerebral infarcts and reduced total gray, white, and hippocampal volumes. Additionally, T2DM-related gray matter loss is primarily concentrated in the medial temporal, anterior cingulate, and medial frontal lobes. The study further demonstrated that T2DM is associated with poorer visuospatial construction, planning, visual memory, and speed (Mehta et al., 2014). This study investigated the relationship between urine albumin-to-creatinine ratio (UACR) and regional brain volumes in type 2 diabetes mellitus patients. It found that UACR is associated with lower gray matter volume and worse executive function, independent of diabetes control and hypertension. Researchers in Hughes et al. (2018) analyzed the connections between cognitive function and brain structure in African Americans with type 2 diabetes. The findings revealed that individuals with smaller gray matter volume and increased white matter lesion volume tended to perform more poorly on global cognitive and executive function tests. These results suggest that there may be a complex relationship between brain health and cognitive performance in diabetic patients. The study in Tejasree and Agilandeeswari (2022) presents a gradient boosting-based ensembled classification technique to classify brain cancer *in vivo* using hyperspectral images. It employs a graph-based clustering approach for feature selection and a multi-scale CNN method for feature extraction. According to the experiment's findings, the proposed model performs better than the Random Forest (RF) and Support Vector Machine (SVM) classification approaches.

Early detection and diagnosis of brain atrophy is important for the best possible treatment and to increase the patient's life span. Traditionally, brain atrophy classification is done by human experts who examine medical images of the brain, such as MRI scans. However, this is a time-consuming and subjective process accounting for the need for an automated computerized model to classify brain atrophy more accurately and efficiently. Deep learning models have shown potential advantages over traditional methods in the medical sector. These models can analyze datasets to identify complex patterns and provide more accurate predictions, which could lead to earlier detection and better management of neurodegenerative disorders. Thus, there is a growing need for a deep learning-based brain atrophy diagnosis model that can enable early detection of neurodegenerative disorders and identify the specific features of a brain image that have high significance for classifying and predicting brain atrophy. This model can give clinicians more insights into the patient's condition and guide treatment decisions. Some potential advantages are as follows: facilitates early detection, improves accuracy and efficiency, classifies different types of brain atrophy, predicts the progression of brain atrophy, and identifies the risk of brain atrophy.

The major contributions of this study are as follows.

- We proposed a Transfer Residual Attention U-Net (TRAU-Net) model based on U-Net and transformer for segmenting the affected brain region's white matter, gray matter, and cerebral spinal fluid.
- A multi-class classification model, Multinomial Logistic Regression with Attention Swin Transformer (MLAST), is proposed to classify the multiple types of brain atrophy.
- A Multivariate CoX Regression (MCR) model is developed for prediction.
- XAI models and *k*-fold cross-validation techniques have been employed to validate the proposed model. Additionally, a comparison with existing systems has been performed to evaluate the effectiveness of the proposed model.

## 2. Proposed methodolgy

### 2.1. Dataset acquisition

Our study gathered 259 patients of T2DM with brain atrophy and without T2DM brain atrophy and normal individuals as subjects. The National Institute on Aging-Alzheimer's Association (NIA-AA) study criteria for probable AD1 were used to diagnose atrophy and Alzheimer's. We defined subjects with normal cognition as those without any history of neurologic or psychiatric disorders; normal cognitive function was determined using neuropsychological tests and diabetic history. The results of our study provide important insights into the diagnosis and management of atrophy disease and may have broader implications for understanding the underlying causes of cognitive decline.

### 2.2. Pre-processing

**Figure 1:**
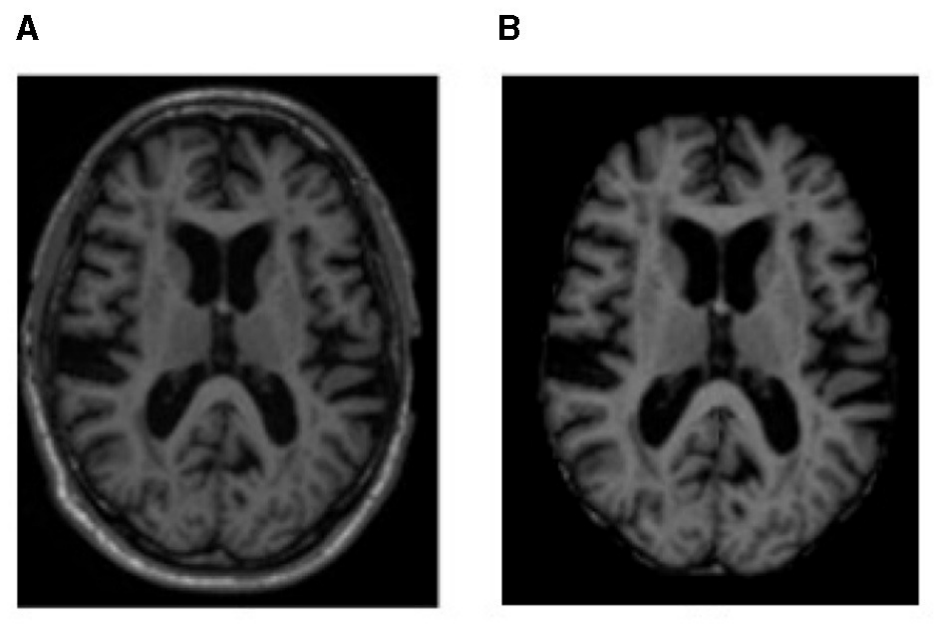
MRI sample representation from the dataset of **(A)** original MRI scan and **(B)** brain region image, i.e., an image after processing with BET to remove non-brain regions.

**Figure 2:**
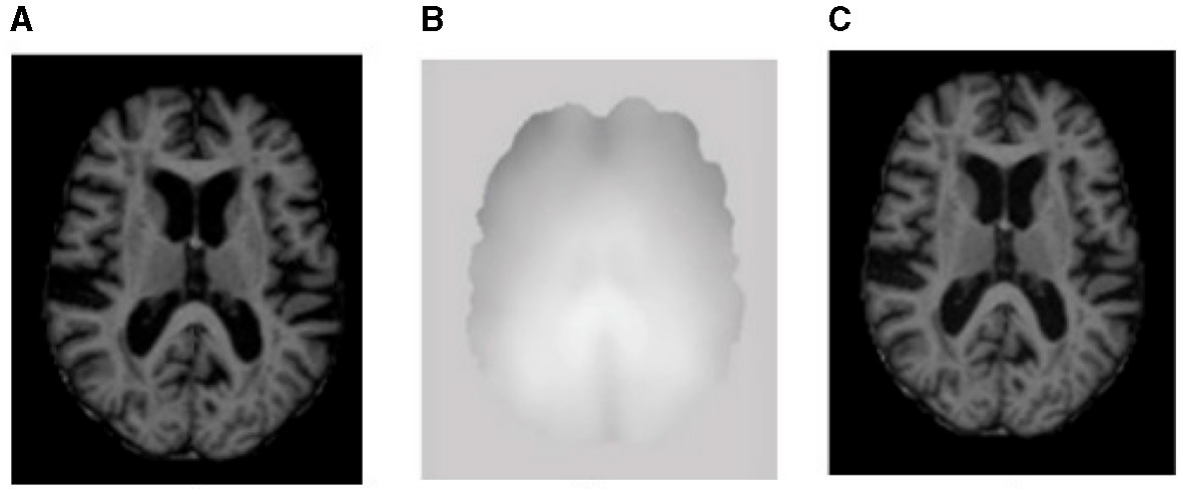
Sample representation of pre-processed images **(A)** brain region, **(B)** bias intensity, and **(C)** bias-corrected image after pre-processing.

After obtaining the MRI dataset, various pre-processing methods are applied to accurately segment the images into different brain tissues, as shown in Figures 1, 2, i.e., brain region extraction, bias correction, and image normalization.

#### 2.2.1. Brain region extraction

Brain MRI scans show components such as tissue, head, eye, fat, spinal cord, and skull. To identify the voxels as either brain or non-brain, skull stripping must be performed; a Brain Extraction Tool (BET) is used for this purpose in our study. The skull-stripped images were obtained using BET with a Fractional Intensity (FI) value of 0.01. The output of skull stripping can be an image containing only brain voxels or a binary value that assigns a value of 1 for brain voxels and 0 for non-brain voxels.

#### 2.2.2. Bias correction

Image contrast due to magnetic field inhomogeneity can be fine-tuned with the help of bias field correction. The bias field depends on the strength of the magnetic field and is almost negligible when the MRI is performed at 0.5 T; when the magnetic field strength is 1.5 T, 3 T, or larger, it is considered strong and can impact the analysis of the MRI. Figure 2B shows the bias field, and Figures 2A, C shows the MRI before and after bias field correction, respectively.

The significant challenge in the quantitative analysis of MRI is obtaining comparable results between consecutive scans, different anatomical regions, and even within the same scan. This is largely due to undesirable signals that must be suppressed before the segmentation. Among these signals, the bias field is one of the most important to eliminate. The bias field is a low-frequency magnetic field variation within the MR images that can cause brightness and contrast irregularities while obscuring fine details. To address this issue, a normalization filter is required to remove the low-frequency bias field and preserve the image's details. This normalization filter optimizes the images' brightness and contrast and regulates the MRI signals' intensity. The filter also improves the accuracy of segmentation algorithms by removing the intensity variation in the image.

A normalization method was employed in this study to remove the bias field present in MRI images. The method utilizes a sliding window approach, where a region is defined within the image, and the bias field is compensated for by analyzing the histogram of pixel values within the region. To calculate the normalization factor, all the pixels within the region of the sliding window (SW) are gathered as input data. The standard deviation (SD) of all the gathered pixel values is then calculated. Additionally, the midline standard deviation (MSD) of the SW is also computed. The pixel data in the mid-line is then shifted to the desired offset where the value of MSD meets that of SD. This ensures that the pixel data compensates for the bias field effect.

To further adjust the pixel values for the bias field effect, the difference between the SD and MSD is added to the pixel values of the mid-line. The minimum and maximum pixel values of the mid-line are then obtained, after which the mid-line pixel data is stretched to its maximum data resolution. The minimum value is set to zero during this process, and the maximum value is set to 255, as demonstrated in the equation below.

(1) $$\begin{array}{l} \;P_{N}=\frac{\left(P-P_{min}\right)\times Pbd_{max}}{P_{max}-P_{min}} \end{array}$$

Where *P* is the pixel value, *P*_*N*_ represents the normalized pixel, *P*_*min*_ is the minimum pixel value, *P*_*max*_ is the maximum pixel value, and the maximum value of pixel bit depth is denoted by *Pbd*_*max*_. By utilizing this method, the algorithm can effectively remove the bias field from the MRI images, leading to more accurate and reliable results. This normalization technique ensures that the images are of the highest possible quality and free from any potential bias, essential for accurate diagnosis and treatment planning in medical imaging.

#### 2.2.3. Image normalization

The Convolutional Normalized Mean Filter (CNMF) is a method for normalizing and enhancing MRI scans, which removes noise from images while preserving their edges. The CNMF algorithm first divides the image into small patches. Then, for each patch, it calculates the median value of the pixels in the patch. The convolved median value then replaces the distorted pixels in the patch. The mathematical formulation of the CNMF algorithm is given below.

(2) $$\begin{array}{l} \;cm_{i}=conv.median\left[I_{i}^{n-1}i\epsilon K.W\right] \end{array}$$

In the above Equation (1) *cm*_*i*_ represents the convolved median value; the convolved median value is obtained through the dot product of convolutional kernel (K) and window size (W), *I*^*n*−*i*^ is the iteration of image sequence i. NMF is used as the backbone for the CNMF model. The main difference between NMF and CNMF is that CNMF can improve image quality without distorting the image information or edges. This is because CNMF uses a convolutional filter to remove noise and apply a convolved filter, as shown in Equation (1). Normalization transforms image I into a normalized image *I*_*N*_ with minimum *Min*_*N*_ and maximum *Max*_*N*_ intensity values, as shown below.

(3) $$\begin{array}{l} \;I_{N}=\left(I-Min\right)\frac{Max_{N}-Min_{N}}{Max-Min}+Min_{N} \end{array}$$

All the scans were randomly divided into a training and test set in an 80:20 ratio, *n* = 188 and *n* = 47, respectively. The initial image correction step is image rotation, assuming each sample is parallel to the horizontal boundaries. Various data augmentation techniques were applied to enhance the training data, including random shifting, random rotation from −5° to 5°, left-right flipping, and random zoom.

### 2.3. Segmentation

For segmentation, we proposed a TRAU-Net (Transformer Residual Attention U-Net) model that provides a residual self-attention process for sequence-to-sequence prediction. TRAU-Net is a hybrid model comprising CNN, UNet, and Transformer to exploit the global context stored by transformers and the detailed high-resolution spatial information from CNN to neutralize the loss of feature resolution caused by transformers. The transformer's encoded self-attentive features are up-sampled and integrated with several CNN high-resolution features to enable accurate localization. The proposed segmentation model for segmenting the brain shrinkage region in MRI scans is developed based upon the TransUNet model in Chen et al. (2021), which consists of an encoder and decoder units (CNN transformer is used as an encoder and UNet decoder as a decoder). The approach involves utilizing an attention transformer model as the backbone of the U-Net architecture, followed by adding residual modules to enhance the performance of the segmentation model. The attention transformer model is a variant of the transformer model, a type of neural network architecture that uses a self-attention mechanism to compute input representations. The attention mechanism allows the model to attend to the relevant parts of the input and ignore the irrelevant ones. In TRAU-Net, the attention transformer model is used to enhance the feature representation of the input, which is then passed to the U-Net architecture.

The encoder unit of the model comprises a residual module with skip connections. This module enhances the feature representation of the input by allowing the model to learn residual mappings between input and output. The output of the residual connection is then passed through a transfer attention mechanism, which aims to identify the critical regions of the image that contain the relevant information for the model to learn. In our work, the brain's gray matter, white matter, and cerebral spinal fluid regions are the regions of interest. The attention mechanism combines feature maps and spatial maps to achieve this objective. The feature maps generated by the convolutional layers are used to compute the attention scores, which measure the importance of each feature map for each spatial location. The spatial maps provide the location information for each pixel and are used to weight the feature maps. The output of the attention mechanism is a weighted feature map that highlights the essential regions of the input image. This map is then passed to the next layer in the network, the decoder path, which is used to reconstruct the output image. Therefore, the proposed TRAU-Net differs from existing U-Net models.

#### 2.3.1. Working

**Figure 3:**
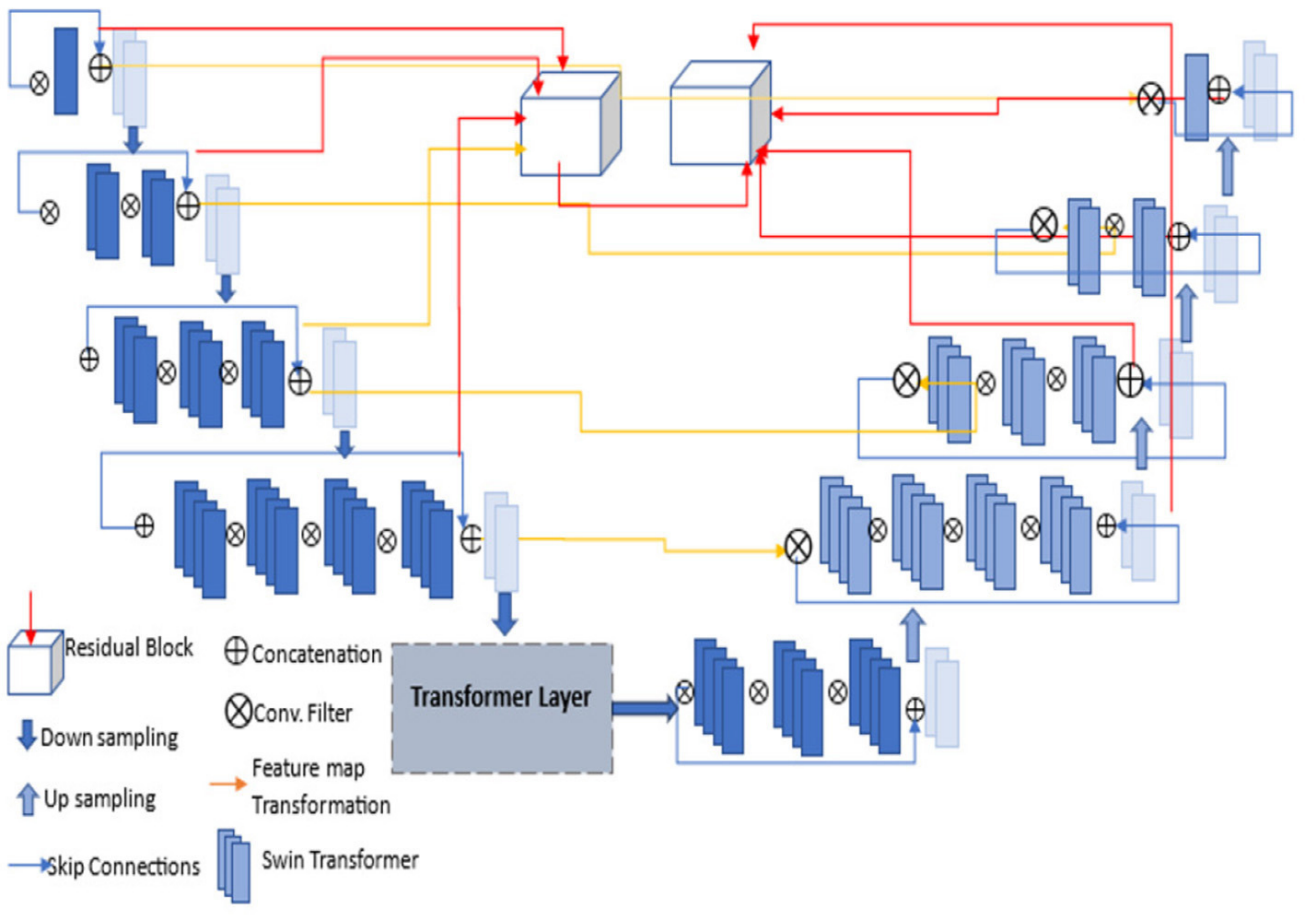
Architecture of proposed TRAU-Net segmentation model; each layer consists of convolutions, activation function (LeakyReLu), down-sampling, and up-sampling. The transformer layer architecture is depicted in Figure 4.

**Figure 4:**
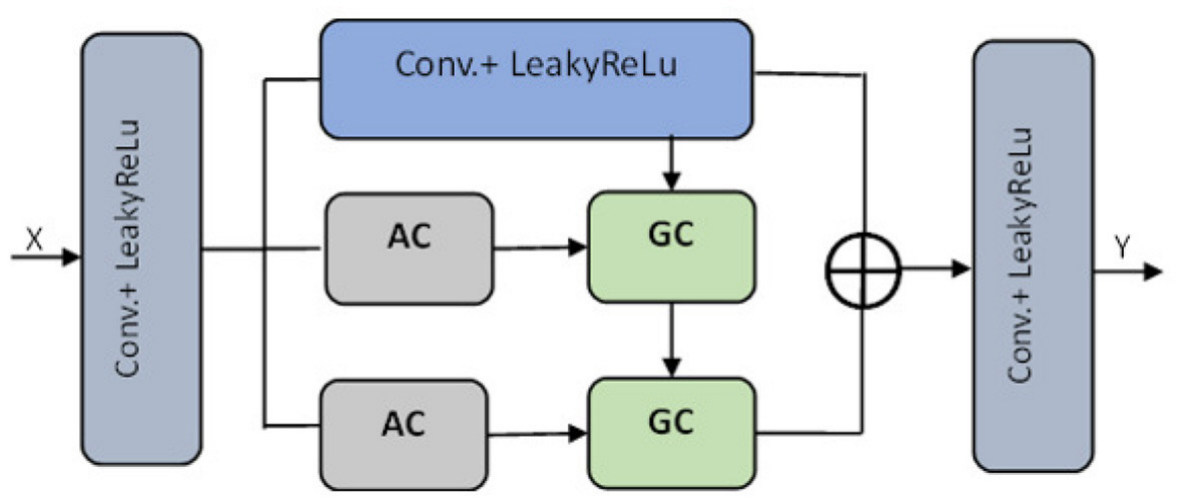
Transformer layer of the segmentation model TRAU-Net [⊕ represents the concatenation, Atrous Convolution (AC), Gated Convolution (GC), Blue lighter conv. block represents 3 × 3, and gray blue block represents 1 × 1].

The pre-processed images are fed into the encoder units to extract higher-level features, and these features are passed to the decoder units, where spatial information is restored. Residual units are employed in the network to increase the number of feature maps produced by convolution operations, as the resolution will decrease and enhance the segmentation result. As seen in Figure 3, the dark blue units represent the encoder. In contrast, the light blue units represent the decoder and skip connections are redesigned for both network pathways. Inter-connection between the single network (an encoder-decoder pathway) with full-length simple skip connections by addressing the issue of U-Net, i.e., a dense and nested skip connection. The TRAU-Net model comprises essential blocks: Atrous Convolution (AC) block, Depth-wise Separable Convolutional (DSA), Gated Convolutions (GC) block, as shown in Figure 4, and residual block. Consider an MRI image *IϵR*^*H*×*W*×*C*^ as spatial image resolutions (H and W are the height and width of the image, and C represents several channels) are given as input to the model, the main objective is to identify the equivalent feature map with *H* × *W* size. In general, learning algorithms like CNN or UNet are trained on the image data, which encodes the image into higher-level features and then decodes to generate the original spatial resolution. The proposed model uses a transformer mechanism to introduce a self-attention model in the encoder unit. The following section explains the working of TRAU-Net briefly.

As seen in Figure 2, the input image I is reshaped through tokenization into a series of two-dimensional flattened sub-regions $I_{S}^{n}\epsilon R^{S^{2}\cdot C};i=1,...,N$. The size of each sub-region is denoted as *S* × *S*, and the series of sub-regions are defined by the following Equation (4).

(4) $$\begin{array}{l} \;N=\frac{HW}{S^{2}} \end{array}$$

Vectorization is performed on the subregions, and the obtained patches are mapped to an *N*-dimensional embedding space through a linear transformation technique. The patch spatial information is encoded, and position embeddings are applied to ensure the spatial information is not lost. These embeddings are added to the patch embeddings, preserving the positional information required for precise analysis. By retaining this information, data is effectively analyzed and interpreted more accurately and efficiently, as explained in Equation (5).

(5) $$\begin{array}{l} \;Y_{0}=\left[I_{S}^{1}E;I_{S}^{2}E;....;I_{S}^{N}E\right]+E_{pos} \end{array}$$

In the above equation, the position embedding is denoted by $E_{pos}=R^{N\times D}$ and the embedding projection of the sub-region is denoted by *EϵR*^(*S*^2^·*C*)×*D*^. Similar to the model in Chen et al. (2021), the encoder of the proposed ATU-Net is a transformer-based U-Net model comprising an attention mechanism in the transformer layers of the encoder to process and transform the sequence data. The encoder consists of a series of Transformer layers, and the attention mechanism is a powerful tool that allows the model to learn long-range dependencies in images by transforming to different parts of the image. Therefore, when the transformer is employed as an encoder, initially, image sequentialization must be performed to convert 3D images to 2D image subregions, as explained in Equation (5). The outcome is then transformed to the transformer layer consisting of atrous attention convolutions (AC) and gated convolutions (GC) blocks, as shown in Figure 4. To acquire features from various receptive fields, the model comprises 1:3 × 3 convolution operation and two AC modules with 3 × 3 convolution rates of two and three, respectively. To guide the discriminative extraction process of the original feature, we input the features acquired by the 3 × 3 conv. and the AC module with an atrous rate of two *r* = 2 into the GC. To further extract the discriminative feature, the feature from the first GC and AC module with a *r* = 3 are re-fed into the GC as illustrated in Figure 4. The decoder of the TRAU-Net model consists of up-sampling layers, normalization layers, ReLU function, skip connections, and a swin transformer. In the decoder stage, the input features are up-sampled and concatenated with the corresponding skip connection feature maps from the encoder stage. This is done in each decoder step to ensure the output is accurate. Once the concatenation is completed, the output is fed into the swin transformer layer for further processing. It produces $\frac{H}{4}\times \frac{H}{4}$ resolution output image, which is down-sampled into *H*×*W* and $\frac{H}{2}\times \frac{H}{2}$ resolution to obtain the low-dimensional features, and finally, the output segmented mask is produced. The final segmented mask will be generated using all these output features combined through a skip connection and softmax function.

### 2.4. Classification

The CT axial images of the brain were analyzed to determine the cortical atrophy level in various brain regions. Specifically, the visual rating of cortical atrophy in the frontal, parietal, and medial temporal lobes was assessed. To evaluate frontal atrophy (FA), the simplified Pasquier scale or global cortical atrophy for the frontal lobe (GCA-F) was utilized. Parietal atrophy (PA) was measured using the axial template of the posterior atrophy scale. Finally, the medial temporal atrophy (MTA) was assessed using the hippocampus and surrounding CSF, demonstrating excellent agreement with Scheltens' coronal visual rating scale5. When evaluating FA and PA, a four-point scale of 0–3 was used. More severe atrophies were measured when there was asymmetry for FA and PA.

In contrast, bilateral atrophies were separately measured for MTA evaluated on a five-point scale of 0–4. These careful evaluations allowed for a detailed understanding of the cortical atrophy level in the brain's various regions. For classification, features such as White Matter (WM), gray Matter (GM), and Ventricular Size (VS) from the segmented masks were used as described in Table 1. Periventricular hyperintensity (PVH), FA, and MTA are the most common atrophy measures in diagnosing atrophy and other brain disorders. Extracting the features is difficult. Therefore, the Global Cortical Atrophy (GCA) measure was used, which provided valuable insights for the diagnosis.

**Table 1:** Description of features acquired from segmented images for multi-class classification.

**Features**		**Description**
Volumetric ratio	GMV	Volumetric ratio of gray matter in the whole brain
	WMV	Volumetric ratio of white matter in the whole brain
	GMWMV	The sum of the volumetric ratio of gray and white matter in the whole brain
	VSV	Total number of ventricle voxels in the whole-brain volume's
Area ratio	GMA	Area ratio of gray matter in a particular region
	WMA	Area ratio of white matter in a particular region
	GMWMA	The sum of the area ratio of gray and white matter in a particular region
	VSA	Pixel number of ventricle voxels in the particular region

**Figure 5:**
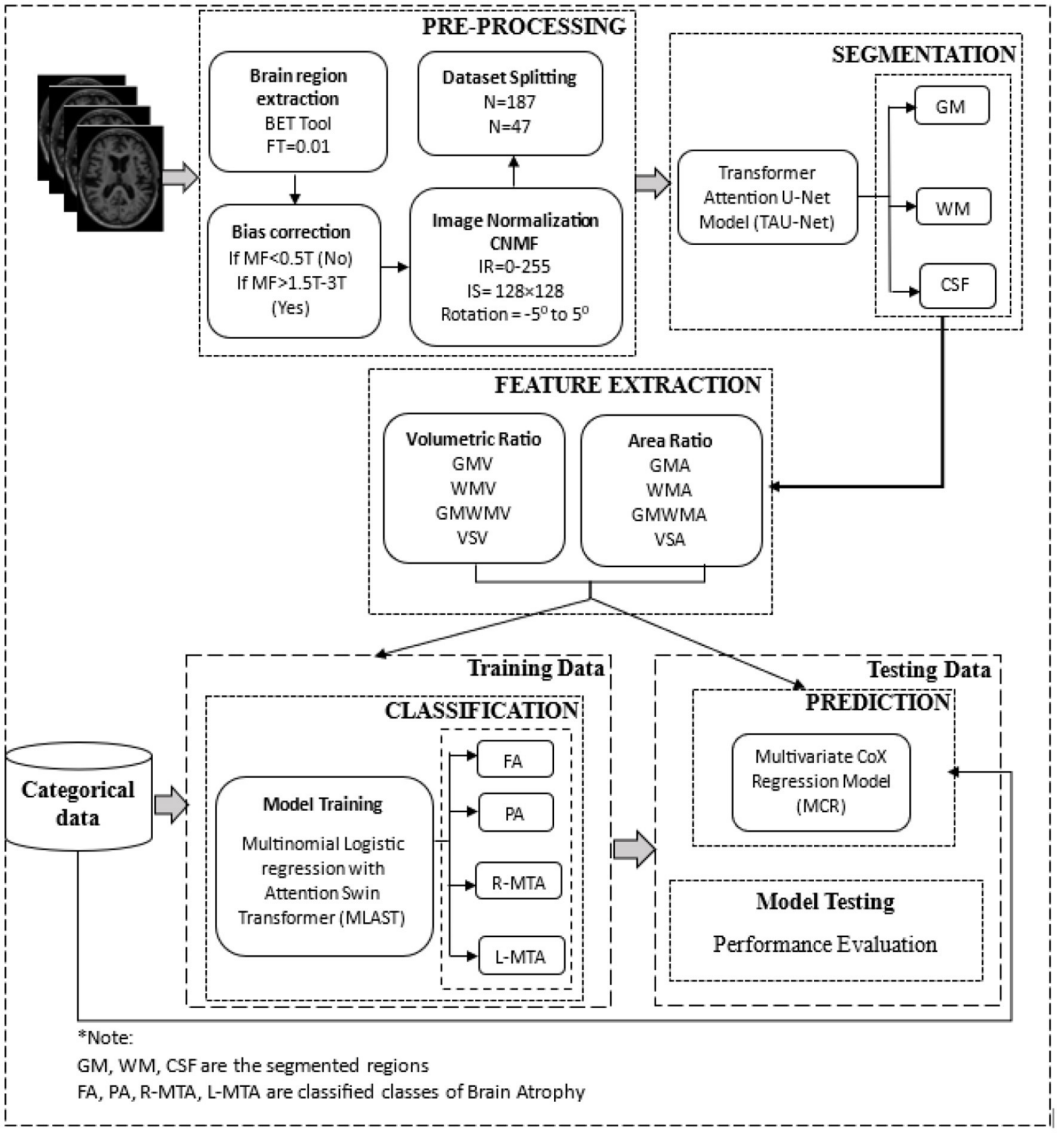
The overall architecture of the proposed deep learning-based brain atrophy diagnosis model. Comprising four stages: pre-processing, segmentation, feature extraction, and classification using brain MRI. MRI scans are pre-processed to remove the non-brain tissues and then segmented using the proposed TRAU-Net model during the pre-processing stage. Features are extracted from the segmented regions and fed as input to the classification model MLAST along with categorical data for multi-class classification of BA, i.e., FA, PA, R-MTA, and L-MTA.

After extensive research, the presence of atrophy was classified using the output of the segmentation model and the features described in Table 1. As shown in Figure 5, the categorical data is also used to train the classification model, such as age, gender, fasting glucose, systolic and diastolic blood pressure, body mass index (BMI), weight, smoking history, insulin usage, high cholesterol, and duration of DM. A Multinomial Logistic regression with Attention Swin Transformer (MLAST) model was proposed for classification. The analysis involved training a Multi-nominal Logistic Regression (MLR) classifier with an attention swin transformer to differentiate between these classes, which proved highly effective. Furthermore, the age, gender, and medical history of diabetic and non-diabetic patients were incorporated into the model to develop a more comprehensive and integrated approach. These findings demonstrate the potential of this approach as a valuable tool for early detection and diagnosis of atrophy-related conditions. The MLR model was ensembled with the proposed TRAU-Net model as the classifier to classify the atrophy regions. The key difference between MLAST and TRAU-Net models is the convolutional swin transformer with MLR is used after the encoder model instead of a decoder of TRAU-Net, which employs a swin transformer to classify the BA. In the decoding path, the convolutional transformer with MLR uses intermediate high-resolution CNN feature maps to enhance model performance. To inherently deal with multi-class classification issues, the multinomial logistic regression technique extends the logistic regression model by changing the loss function to cross-entropy loss and the predicted probability distribution to a multinomial probability distribution.

### 2.5. Prediction

Chronological age prediction for the proposed model is made by computing the features of all the images and segmented labels. ROI mean intensity value of each image type and WM, GM, and CSF volume are also used to predict the chronological age. Therefore, eight features are considered, comprising eight mean intensity values and six volumes. High-order Features (HoF) were not considered as they resist changes in basic image features, making them difficult to predict. Multivariate Cox Regression (MCR) model was used to predict the chronological age of T2DM patients with BA. The MCR model is a statistical approach considering semi-parametric distributions for survival time predictions over multiple predictors mathematically (Christensen, 1987) defined as follows.

(6) $$\begin{array}{l} \;z\left(t\right)=z_{0}\left(t\right)*e^{\sum x_{i}*\beta_{i}} \end{array}$$

The study estimates the probability of occurrences before a certain point in time using the hazard function *z*(*t*) based on a random hazard model at time *t*. The exponential function of the hazard model ∑*x*_*i*_ ∗ β_*i*_. It is computed while all independent variables are nullified, and the regression factor β of x is considered. Only features with a probability *p* < 0.05 were considered, while those with *p* > 0.05 were eliminated. After finalizing the features, a logistic regression model was used to predict the survival rate, and the accuracy was evaluated using a validation set and leave-one-out cross-validation. The model performance was measured using correlation coefficient (*r*), coefficient determination *r*^2^, and Mean Square Error (MSE); the obtained outcome for the prediction model is presented in Table 4.

### 2.6. Experimental results

**Figure 6:**
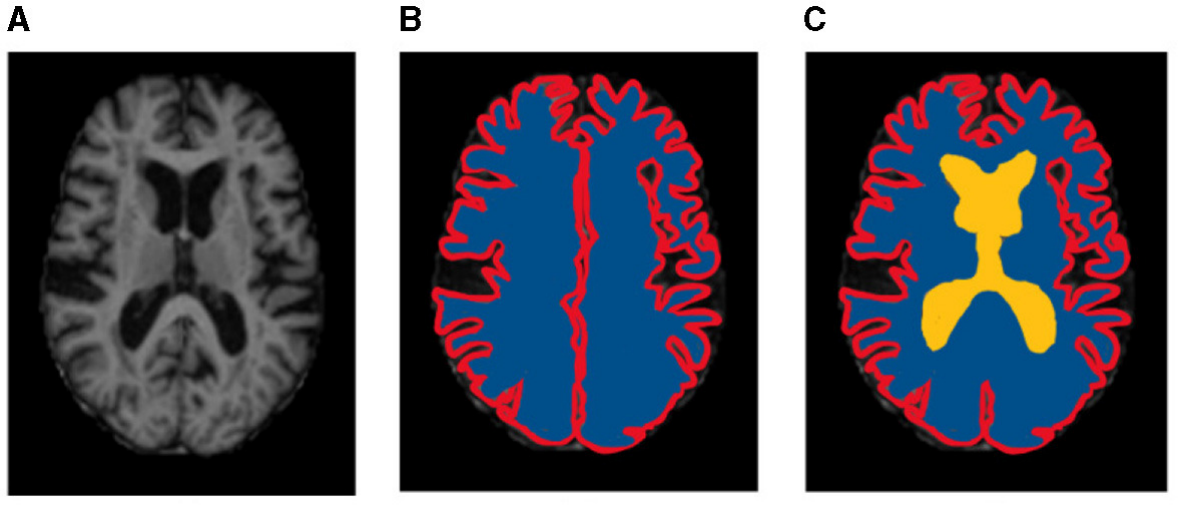
Sample representation of segmented images **(A)** pre-processed input image to segmentation model, **(B)** Global Cortex Atrophy (GCA), and **(C)** GM, WM, & CSF [red represents gray matter (GM), blue represents white matter (WM), and yellow represents cerebral spinal fluid (CSF)].

Results of segmentation from the proposed TRAU-Net model for people with BA are shown in Figure 6. As discussed in the above segmentation and classification sections, the GCA is closely associated with diagnosing BA and other brain disorders. Dice Similarity Co-efficient (DSC) was calculated for every subject, and the average was 0.9154 ± 0.0132. It was observed that the proposed segmentation model has outperformed the existing models. Additionally, the MRI image was segmented into three regions: white matter (WM), gray matter (GM), and Cerebral Spinal Fluid (CSF), as shown in Figure 6C; red represents GM, blue represents WM, and yellow region represents CSF, for evaluation of segmentation model three different measures were applied DSC, Absolute Volume Difference (AVD), and Hausdorff Distance (HD). The similarity between a predicted segmentation mask (S) and the actual segmentation mask/ground truth mask (G) can be measured using the Dice score, DSC. A score of 1 shows complete overlap, while a 0 indicates no overlap; mathematically, it is defined as in Equation (7).

(7) $$\begin{array}{l} \;DSC=\frac{2|G+S|}{\left|G|+|S\right|} \end{array}$$

The AVD is the ratio of the difference between the *V*_*S*_ *V*_*G*_ segmentation volume to the ground truth mask *V*_*G*_. A lower AVD value indicates a more accurate segmentation, mathematically defined as in Equation (8).

(8) $$\begin{array}{l} \;AVD=\frac{V_{S}-V_{G}}{V_{G}} \end{array}$$

The Hausdorff distance determines the distance between segmentation results and actual data. A smaller value of HD indicates that the ground truth and segmentation results are closer together, which translates to a higher level of accuracy in the segmentation process; Equation (9) represents the mathematical formula to calculate HD.

(9) $$\begin{array}{l} \;HD=mx\left\{h_{95}\left(S,G\right),h_{95}\left(G,S\right)\right\} \end{array}$$

**Figure 7:**
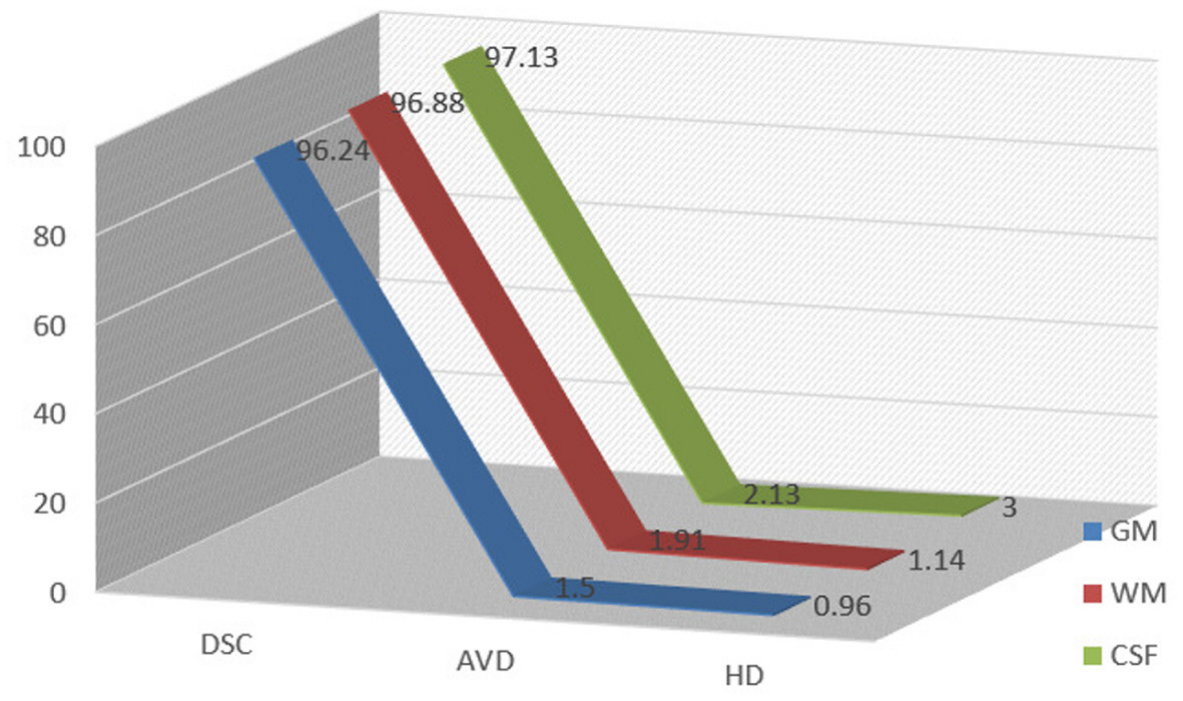
Performance measures obtained for segmentation models in terms of DSC, AVD, and HD.

Table 2 demonstrates the results of the segmentation model, and the graphical representation of acquired results is illustrated in Figure 7. Figure 6 represents the ground truth image and segmentated image, differentiating three categories, i.e., WM, GM, and CSF. The proposed classification model was also applied for segmentation but didn't achieve performance compared to TRAU-Net. Similarly, the segmentation model is applied to perform the multi-class classification task.

**Table 2:** Performance measures and achieved results for the proposed segmentation model TRAU-Net.

**Brain region performance measure**	**GM**	**WM**	**CSF**
DSC	0.9624	0.9688	0.9713
AVD	1.50	1.91	2.13
HD	0.96	1.14	1.05

Different performance measures were applied to evaluate the proposed classification model's performance: accuracy, precision, recall, f1-score, and ROC-AUC curve, as illustrated in Table 3. A confusion matrix is a matrix that displays a machine learning model's performance on a set of test data. It is frequently used to assess how well categorization models work. These models try to predict a category label for each input event. The matrix shows how many true positives (TP), true negatives (TN), false positives (FP), and false negatives (FN) the model generated using the test data. Classification accuracy refers to the proportion of correct predictions compared to the total data. Computation of accuracy is done by using Equation (10).

(10) $$\begin{array}{l} \;A=\frac{TP+TN}{TP+TN+FP+FN} \end{array}$$

Precision is essentially the proportion of positive samples correctly categorized as true positives out of the total number of samples classified as positive. Equation (11) is used to calculate the precision.

(11) $$\begin{array}{l} \;P=\frac{TP}{TP+FP} \end{array}$$

The recall is determined as the proportion of Positive samples properly identified as Positive to all Positive samples, as illustrated in Equation (12). The recall measures how well the model can identify positive samples. A higher recall signifies that the model has correctly identified many positive samples.

(12) $$\begin{array}{l} \;R=\frac{TP}{TP+FN} \end{array}$$

An alternative machine learning assessment statistic called the F1 score evaluates a model's predictive ability by focusing on its performance inside each class rather than its overall performance, as is done by accuracy. F1 score combines a model's accuracy and recall scores, two measures that compete with one another. It is defined as the harmonic mean of precision and recall, as shown in Equation (13).


(13) $$\begin{array}{l} \;F1=\frac{2\times \left(P\times R\right)}{P+R} \end{array}$$

**Table 3:** Illustration of achieved performance of proposed classification model for brain atrophy types FA, PA, R-MTA, and L-MTA in terms of accuracy, precision, recall, F1-score, and AUC measured in % (frontal atrophy, parietal atrophy, right-medial temporal atrophy, and left-medial temporal atrophy).

**Brain atrophy type performance measure**	**FA**	**PA**	**R-MTA**	**L-MTA**
Accuracy	0.9632 ± 1.325	0.9677 ± 1.912	0.9682 ± 1.715	0.9521 ± 1.877
Precision	0.9688 ± 1.011	0.9717 ± 0.983	0.9791 ± 1.012	0.9583 ± 1.247
Recall	0.9487 ± 1.114	0.9639 ± 2.008	0.9581 ± 1.304	0.9331 ± 1.248
F1-score	0.9586 ± 0.814	0.9676 ± 1.267	0.9684 ± 0.026	0.9455 ± 0.953
AUC	0.9856 ± 0.741	0.972 ± 0.026	0.9824 ± 1.082	0.9637 ± 1.037

The AUC-ROC curve is a statistical measure used to evaluate the performance of a classification model across different threshold levels. The Receiver Operating Characteristic (ROC) curve is a useful tool for evaluating the performance of a classification model. It plots the True Positive Rate (TPR) against the False Positive Rate (FPR) as illustrated in Equations (14) and (15), respectively, at different threshold levels. This graph helps to visualize how well the model can distinguish between the positive and negative classes and can be used to calculate the area under the curve (AUC) to quantify the model's overall performance. By analyzing the ROC curve, we can determine the optimal threshold level that maximizes the model's accuracy and minimize errors.

(14) $$\begin{array}{l} \;TPR=\frac{TP}{TP+FN} \end{array}$$

(15) $$\begin{array}{l} \;FPR=\frac{FP}{FP+TN} \end{array}$$

**Figure 8:**
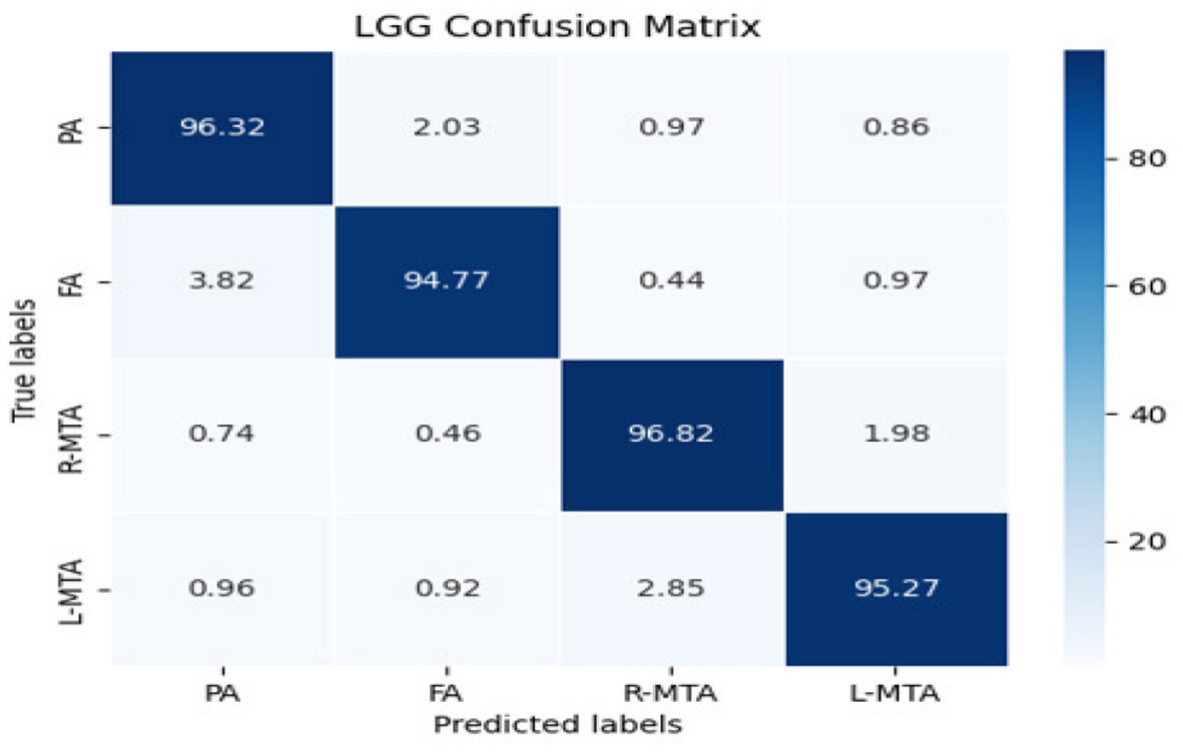
Confusion matrix of the classification model for classifying the types of brain atrophy: FA, PA, R-MTA, and L-MTA.

The confusion matrix shows the number of diabetic patients diagnosed with various types of brain atrophy, FA, PA, R-MTA, & L-MTA, and vice versa. Figure 8 illustrates the confusion matrix for the highest and lowest accuracy outcomes for R-MTA & PA with 0.9682 and 0.9477%, respectively. The main reason for the lower accuracy is the misclassification of label FA labels PA, L-MTA, and R-MTA. Similarities among the atrophy types cause this; therefore, classification is difficult. However, the overall fault diagnosis findings demonstrate that the proposed categorization system can accurately identify different forms of atrophy in diabetic individuals.

**Figure 9:**
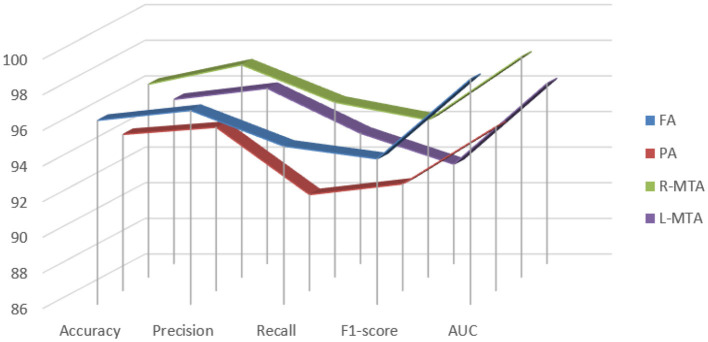
Graphical representation achieved performance measures for the proposed classification model of brain atrophy.

**Figure 10:**
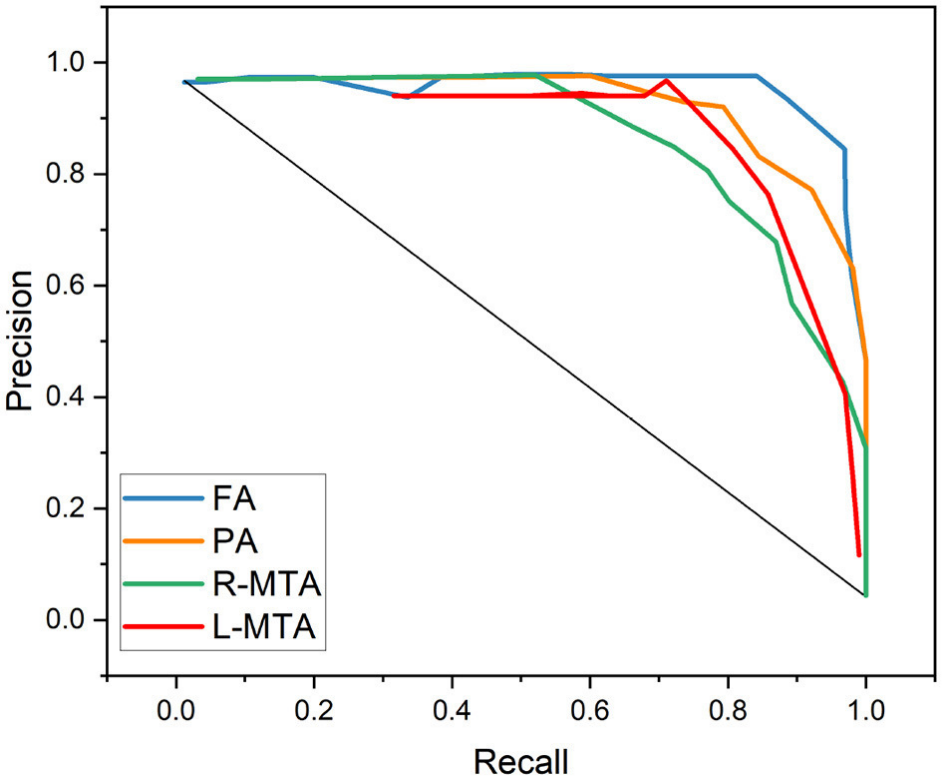
Graphical representation achieved performance measures for the proposed classification model in terms of precision and recall.

**Figure 11:**
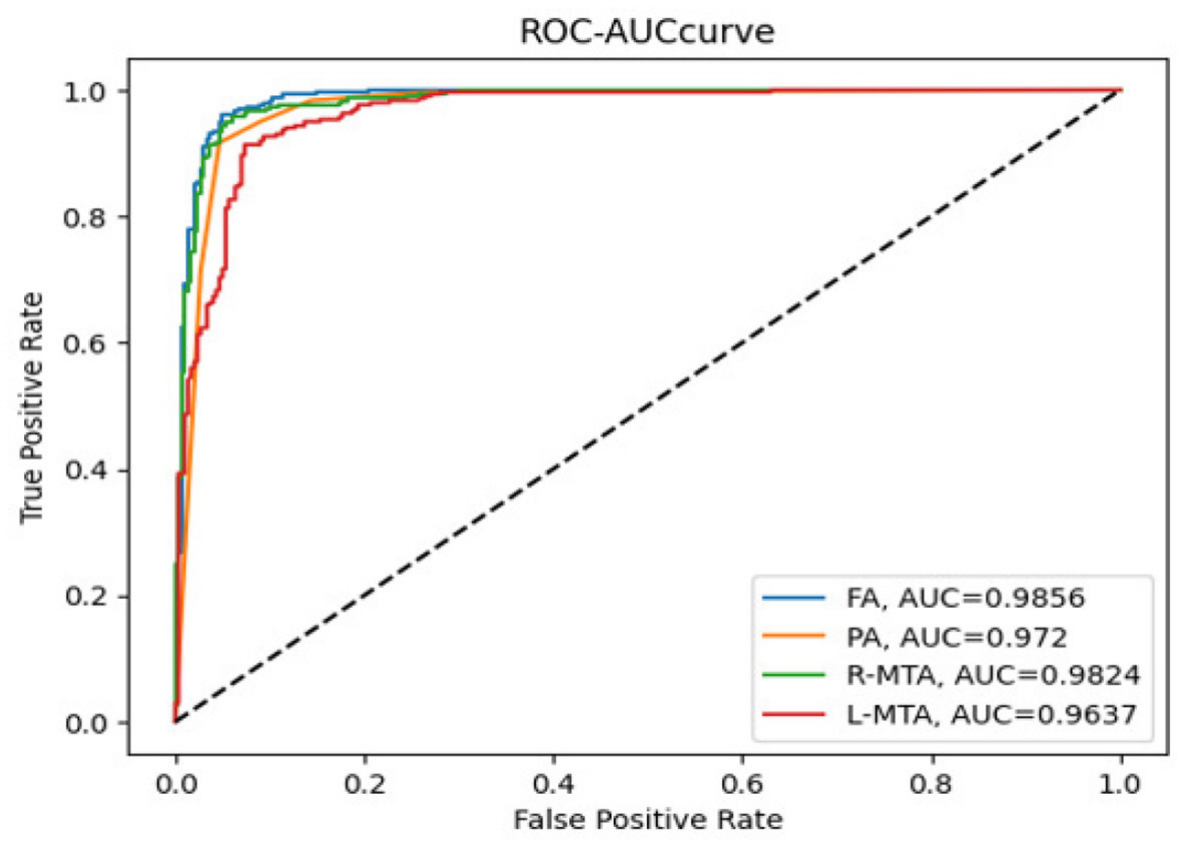
Receiver operating curve along with area under curve generated by proposed classification model for multi-classification of brain atrophy types FA, PA, R-MTA, and L-MTA.

Various performance measures are used to evaluate the classification model, such as confusion matrix, accuracy, precision, recall, f1-score, and AUC-ROC curve. Table 3 and Figure 9 represent the achieved classification results. It is observed from the above table that R-MTA BA type achieved the highest accuracy 0.9682 ± 1.715, followed by 0.9632 ± 1.325 for FA, 0.9521 ± 1.877 for L-MTA, & PA recorded 0.9477 ± 1.912 of accuracy. Figure 10 represents the precision-recall curve. The highest AUC value was recorded for FA 0.9856 ± 0.741, followed by R-MTA 0.9824 ± 1.082, 0.9637 ± 1.037 for L-MTA, & 0.972 ± 0.026 for PA as illustrated in Figure 11.

The outcome of the segmentation model TRAU-Net and the features extracted from the segmented ROI are used to predict the chronological age as explained in the prediction section. various performance measures are used to validate the model, as described in Table 4.

**Table 4:** Performance measures in terms of *r*, *r*^2^, MSE, and accuracy for the chronological age prediction.

**Measure**	***r***	***r* ^2^**	**MSE**
Testing data	0.951	0.904	0.5172
Validation data	0.937	0.878	0.7358
Training data	0.96	0.922	0.7496

To demonstrate that the proposed segmentation and classification models are more advanced versions of existing models, a thorough comparison is conducted between the proposed and existing models using the same datasets, similar parameters, and the same platform. The proposed segmentation model is compared with UNet (Montaha et al., 2023), 2DUNet (Webber et al., 2022), residual UNet (Kermi et al., 2019), and 3DUNet (Oktay et al., 2018) as described in Table 5. As the base models for the proposed classification model are CNN and transformer, the performance of the classification model is compared with 3DCNN (Bäckström et al., 2018), vision transformer (Dosovitskiy et al., 2020), diffusion kernel attention transformer (Zhang J. et al., 2022), and swin transformer (Hatamizadeh et al., 2021) as illustrated in Table 6. Furthermore, the segmentation model is validated using XAI approaches to verify its performance and effectiveness.

**Table 5:** Comparison of proposed segmentation model with existing segmentation models.

**S. no**	**Method**	**Dice score**
1	UNet (Montaha et al., 2023)	93.9
2	2DUNet (Webber et al., 2022)	91
3	Residual UNet (Kermi et al., 2019)	86.7
4	3D UNet (Ballestar and Vilaplana, 2020)	83.16
5	3D UNet (Oktay et al., 2018)	93.41
6	Proposed model	96.24

**Table 6:** Comparison of proposed multi-class classification model with existing models.

**S. no**	**Method**	**Dice score**
1	3D CNN (Bäckström et al., 2018)	90
2	Vision transformer (Dosovitskiy et al., 2020)	89.2
3	Diffusion kernel attention transformer (Zhang J. et al., 2022)	95.1
4	TransBTS (Wang et al., 2021)	90.98
5	Swin transformer (Hatamizadeh et al., 2021)	85.3
6	Proposed model	96

#### 2.6.1. Cross validation

**Figure 12:**
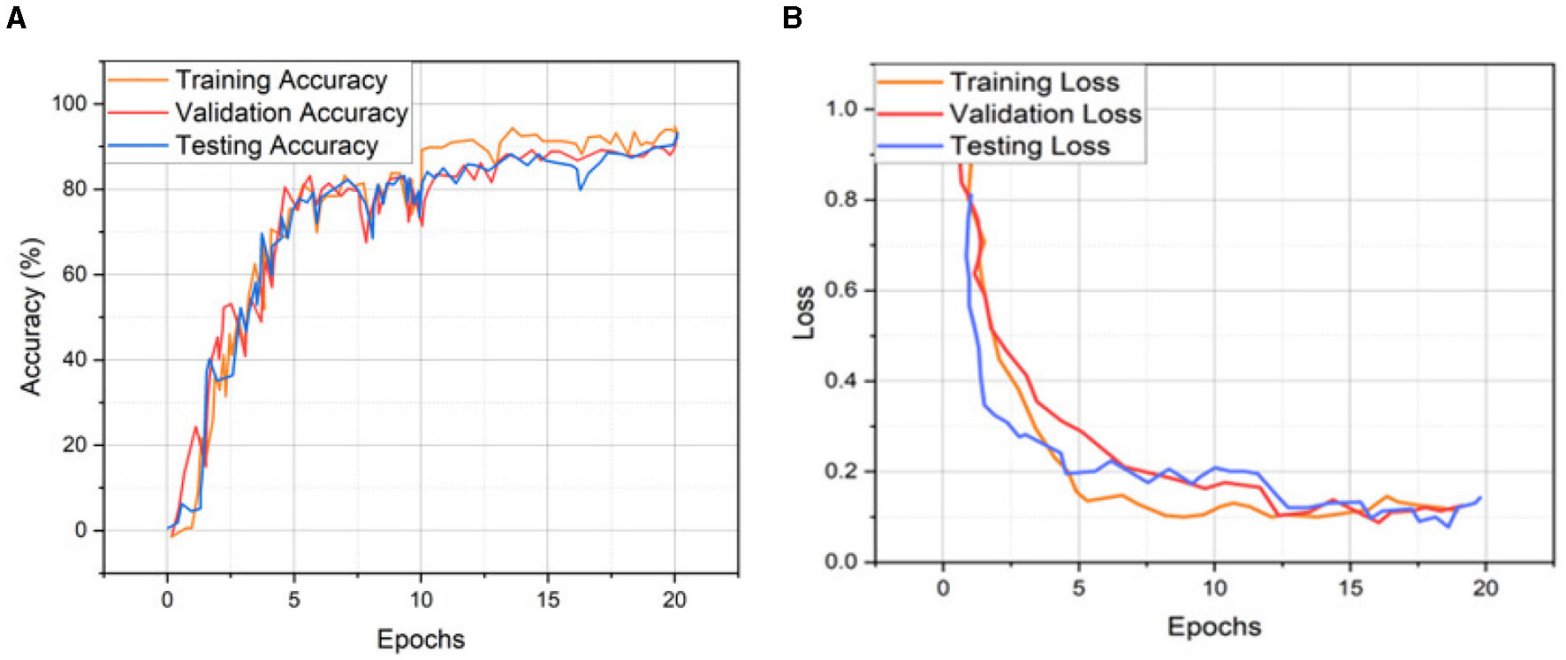
Training, validation, and testing **(A)** accuracy and **(B)** loss for 5-fold cross validation.

Cross-validation (CV) evaluates the model's performance by dividing the dataset into multiple folds and training the model on different combinations. The model's performance can then be evaluated on the remaining fold. CV helps to ensure that the model is generalizing well and producing accurate segmentations on unseen data. CV helps ensure that deep learning models for medical images are reliable and generalizable, essential for accurate decisions and diagnosis. Therefore, our study applied 5-fold cross-validation to validate the proposed segmentation and classification models. In 5-fold validation, the dataset is divided into five subsets known as folds, and then the training is performed on all the subsets except one (5-1). The remaining subset is then used for evaluating the trained model. This process is repeated five times with a different subset reserved for testing each time. Table 7 represents the recorded validation performance of the segmentation model. Overall classification model accuracy and loss of training, validation, and testing datasets are depicted in Figure 12 and Table 8. Table 9 presents the validation performance of the classification model for each fold obtained for each class of brain atrophy FA, PA, R-MTA, and L-MTA.

**Table 7:** Five-fold performance measure for proposed segmentation model.

**Performance → **	**DSC**			**AVD**			**HD**		
**Folds ↓**	**GM**	**WM**	**CSF**	**GM**	**WM**	**CSF**	**GM**	**WM**	**CSF**
K1	0.9243	0.9456	0.9359	1.54	1.88	2.47	1.58	1.08	0.96
K2	0.9461	0.9182	0.9614	1.47	1.95	2.21	1.72	1.13	1.78
K3	0.9275	0.9338	0.9407	1.52	1.90	2.50	1.00	1.57	1.15
K4	0.9384	0.9392	0.9423	1.49	1.93	2.19	0.98	2.42	0.92
K5	0.9338	0.9445	0.9330	1.53	1.86	2.73	1.26	2.21	1.06
Average	0.9340	0.9362	0.9426	1.51	1.904	2.42	1.308	1.682	1.174

**Table 8:** Five-fold performance measure for proposed classification model.

**Performance → **	**Accuracy**			**Loss**		
**Folds ↓**	**Training**	**Validation**	**Testing**	**Training**	**Validation**	**Testing**
K1	0.9489	0.9235	0.9134	0.1414	0.1694	0.0917
K2	0.9246	0.9477	0.9478	0.0763	0.1247	0.1526
K3	0.9431	0.9118	0.9509	0.1352	0.1506	0.1537
K4	0.9357	0.9415	0.9384	0.1736	0.1749	0.1173
K5	0.9694	0.9663	0.9391	0.0962	0.1288	0.0857
Average	0.9443	0.9382	0.9379	0.1245	0.1496	0.1202

**Table 9:** Proposed model performance for five-fold cross-validation technique using various measures precision, recall, F1-score, and AUC for multi-class classification of brain atrophy.

**Performance → **	**Precision**				**Recall**				**F1-score**				**AUC**			
**Folds ↓**	**FA**	**PA**	**R-MTA**	**L-MTA**	**FA**	**PA**	**R-MTA**	**L-MTA**	**FA**	**PA**	**R-MTA**	**L-MTA**	**FA**	**PA**	**R-MTA**	**L-MTA**
K1	0.9419	0.9537	0.9310	0.9411	0.9124	0.9214	0.9334	0.9088	0.9269	0.9371	0.9321	0.9246	0.9897	0.9514	0.9901	0.9332
K2	0.9285	0.9162	0.9681	0.9608	0.9391	0.9570	0.9142	0.9217	0.9328	0.9361	0.9403	0.9408	0.9765	0.9512	1.000	0.9503
K3	0.9127	0.9375	0.9556	0.9374	0.9058	0.9386	0.9199	0.8923	0.9092	0.9547	0.9374	0.9142	1.000	0.9763	0.9864	0.9329
K4	0.9536	0.9584	0.9422	0.9285	0.9218	0.9253	0.9384	0.9001	0.9392	0.9482	0.9206	0.9218	0.9910	0.9619	0.9741	0.9412
K5	0.9445	0.9421	0.9237	0.9240	0.9294	0.9386	0.9285	0.9158	0.9368	0.9403	0.9260	0.9198	0.9826	0.9720	0.9859	0.9534
Average	0.9362	0.9416	0.9441	0.9378	0.9217	0.9362	0.9269	0.9077	0.9289	0.9432	0.9312	0.9242	0.9879	0.9626	0.9873	0.9422

### 2.7. Explainable AI

Explainable AI (XAI) has become increasingly important in domains where critical decisions need to be made, such as in medical image analysis. XAI has demonstrated the black-box approach recognized in DL models, revealing the precise reasoning behind their predictions. As DL models have become more complex, medical experts have been able to understand the results of these models better and use them to quickly and accurately diagnose (Bhandari et al., 2022, 2023; Gaur et al., 2022; Ahmed et al., 2023; Saranya and Subhashini, 2023). To achieve this, two popular XAI algorithms, Shapley Additive Explanation (SHAP) and Local Interpretable Model-agnostic Explanation (LIME), were used in this study.

**Figure 13:**
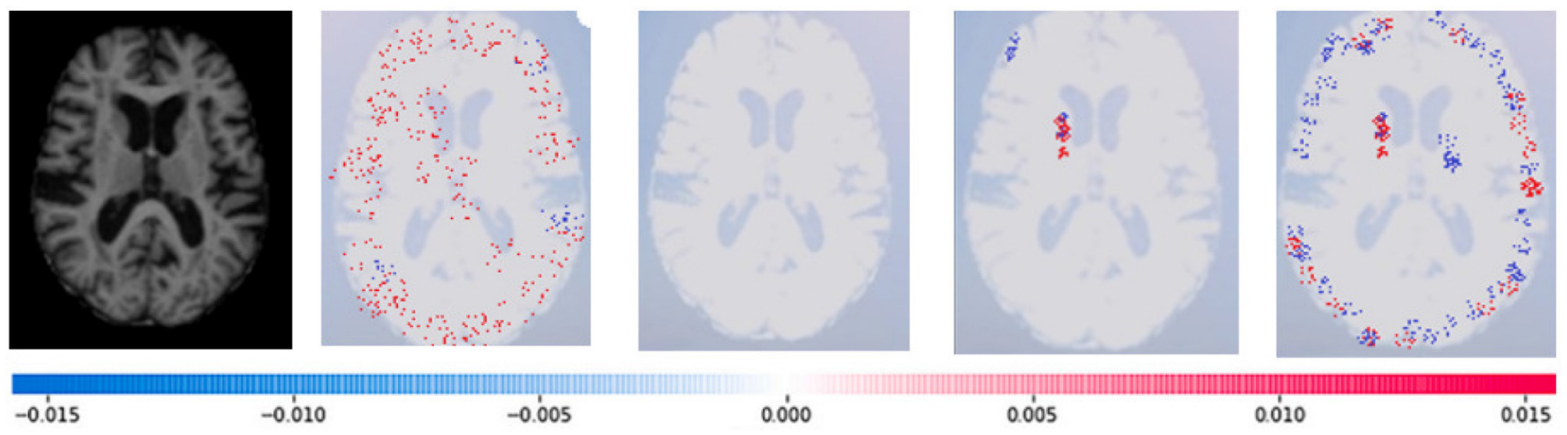
Sample representation of SHAP explainability for the presence of GM (high concentration of red pixels observed in the first explanation image infers that the image indicates the presence of GM).

**Figure 14:**
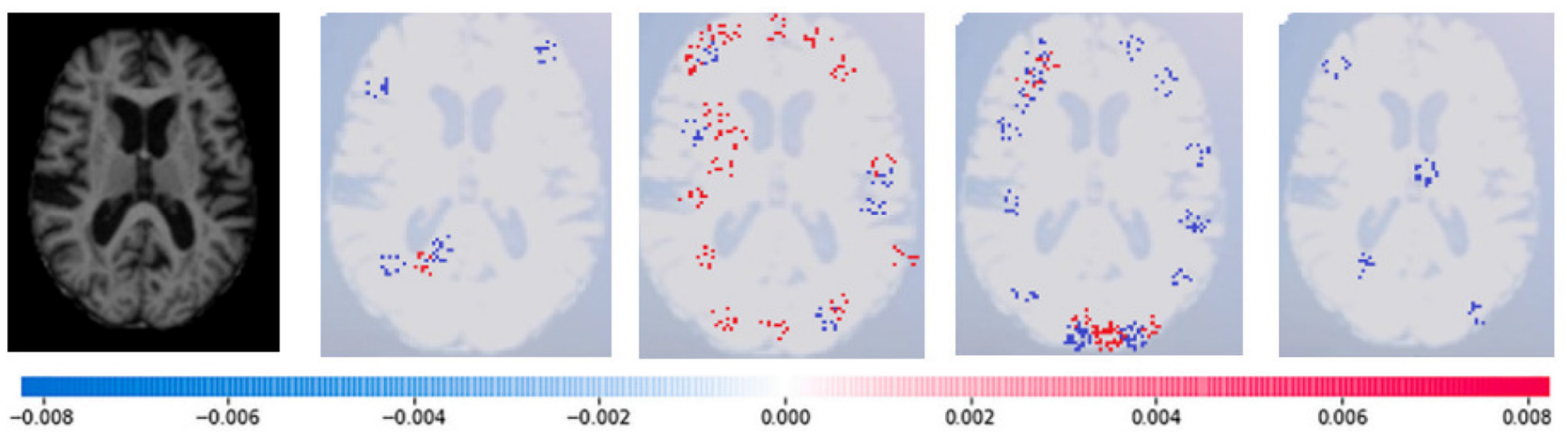
Sample representation of SHAP explainability for normal image (high concentration of red pixels observed in the second explanation image infers that the image is normal).

**Figure 15:**
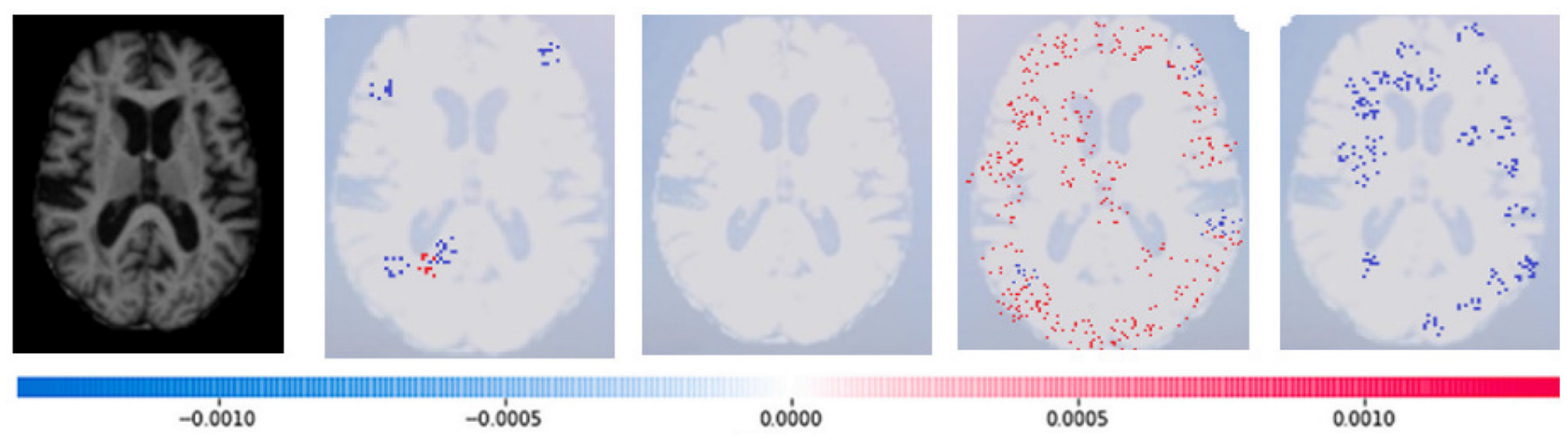
Sample representation of SHAP explainability for WM (high concentration of red pixels observed in the second explanation image infers that the image indicates the presence of WM).

**Figure 16:**
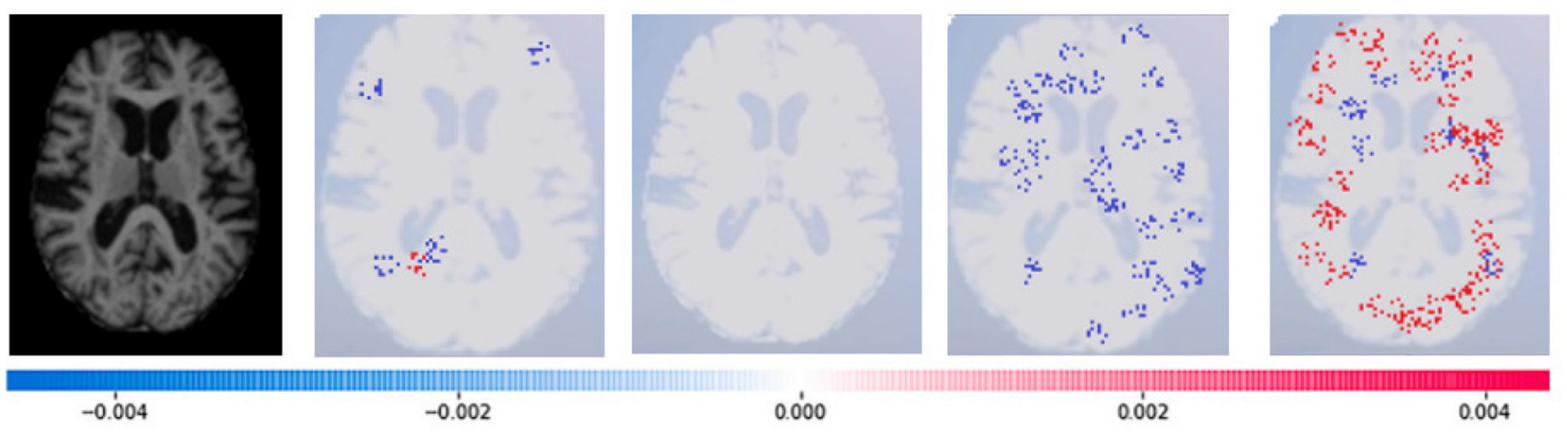
Sample representation of SHAP explainability for CSF (high concentration of red pixels observed in the second explanation image infers that the image indicates the presence of CSF).

**Figure 17:**
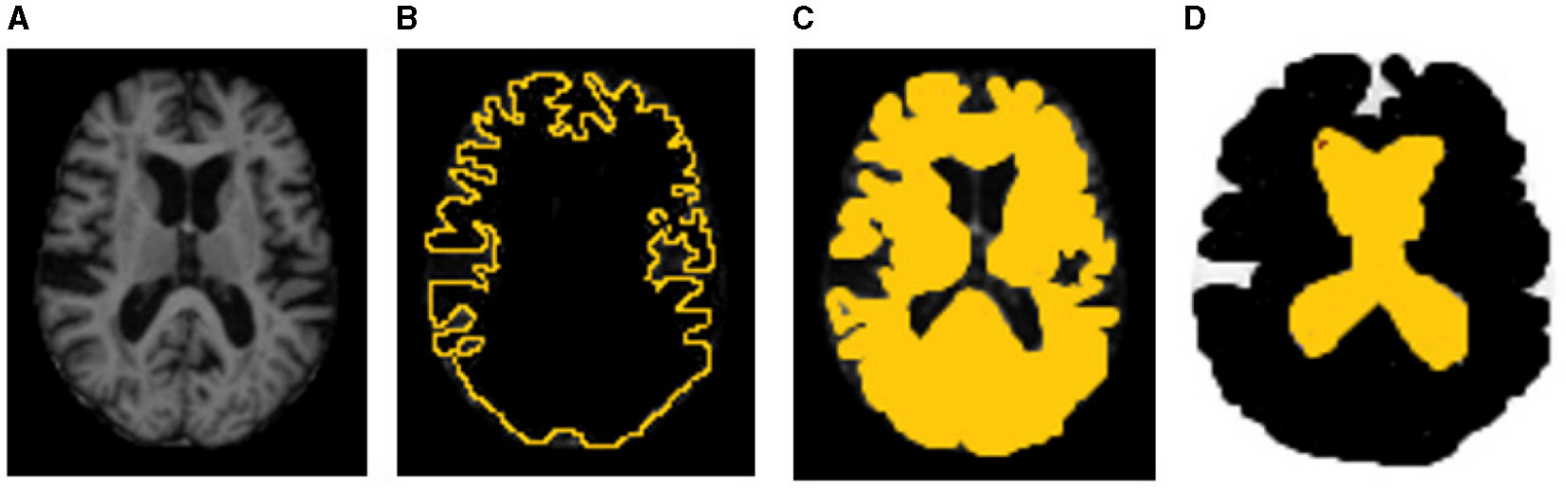
Sample representation of LIME explanability. **(A)** Original input image, **(B)** GM, **(C)** WM, and **(D)** CSF (the highlighted part indicates the LIME regional explanation for GM, WM, and CSF, respectively).

1. SHAP: To evaluate the impact of the model, SHAP employs a technique known as normalizing the marginal feature values. This technique assigns scores to each pixel in an image to exemplify their significance in predicting the class of the image. These scores are then used to substantiate the classification of the image. To obtain the shapley value for each feature, all possible combinations of characteristics of brain atrophy are considered. The shapley value for each feature is computed as the average marginal contribution of the feature across all possible coalitions of features that include the feature. Once the shapley values are computed, they are converted into pixel values, where red pixels indicate an increase in the likelihood of predicting a class. In contrast, blue pixels indicate a reduction in the likelihood, as demonstrated in Figures 13–16. This conversion of shapley values to pixel values allows us to easily visualize the region of interest most significantly predicting a particular class. The following Equation (16), is used to generate the shapley values.
  (16) $$\begin{array}{l} phi_{i}=\sum_{S\subseteq \frac{N}{\left\{i\right\}}}\frac{\left|S\right|!(M-\left|S\right|-1)!}{M!}[f_{x}(S\cup )\left\{i\right\}-f_{x}(S)] \end{array}$$
  Where, for a particular feature, *I, S, and N* are the feature subsets, *f*_*x*_ do shapely values generate the output, $\frac{|S|!\left(M-|S|-1\right)!}{M!}$ represents the weighing factor, which calculates the number of ways the subset S can be permutated, and the predicted result is denoted by *f*_*x*_(*S*) calculated as follows
  (17) $$\begin{array}{l} f_{x}\left(S\right)=E\left[f\left(x\right)|x_{S}\right] \end{array}$$
  To perform the replacement of each original trait *x*_*i*_ using SHAP, a binary variable $z_{b}^{‘}$ is introduced to represent whether *x*_*i*_ is absent or present, as illustrated in Equation (18)
  (18) $$\begin{array}{l} \;g\left(z^{‘}\right)=\phi_{0}+\overset{M}{\underset{i=1}{\sum }}\phi_{i}z_{i}^{‘}=\sum FC+B \end{array}$$
  Where *g*(*z*^‘^) is the local surrogate model, where feature i is derived from the output, and ϕ_*i*_ aids in the comprehension of the model. The SHAP results explain the four output classes—GM, Normal, WM, and CSF—for individual Brain Atrophy cases. The input images are presented on the left-hand side of all figures. In Figure 13, the first explanation image exhibits red pixels that increase the probability of predicting the input image as GM. In contrast, the Normal and WM explanations lack red or blue pixels. The last image in the cluster displays blue pixels, effectively decreasing the likelihood of the input image being identified as CSF. Figure 14 highlights the absence of red pixels in the GM and CSF explanations, a significant number of blue pixels in the WM explanation, and a concentration of red pixels in the Normal explanation. These findings are vital in identifying the image as Normal and provide a deeper understanding of the image features. Similarly, Figure 15, on the other hand, focuses on the WM and highlights the areas where the model identifies the features of WM. Finally, Figure 16 provides a more in-depth view of the CSF. The SHAP result highlights the areas where the model identifies the features of the infection, which is crucial for accurate diagnosis and treatment.
2. LIME: Locally Interpretable Model Agnostic Explanations (LIME) explains any black box machine learning model. It creates a local, interpretable model to explain each prediction. This technique is independent of the original classifier and works locally, explaining the prediction relative to each observation. LIME fits a local model using sample data points similar to the explained observation. The local model can be from the class of interpretable models such as linear models, decision trees, etc. Finally, the explanations provided by LIME for each observation x are obtained by fitting a linear model around the observation as explained in Equation (19).
  (19) $$\begin{array}{l} \;\phi \left(x\right)=argmin_{g\epsilon G}L\left(f,g,\pi_{x}\right)+\Omega \left(g\right) \end{array}$$
  Where *g* represents the explainable model for the sample *x*, which is intended to minimize a loss function *L* while also measuring the proximity of its interpretations to the predicted value of the initial model *f*. The process is carried out while ensuring that the complexity of the model, represented by Ω(*g*), is kept to a minimum. For this purpose, we consider G the set of realizable explanations, which may include decision tree models in a hypothetical scenario. The measure of closeness, π_*x*_, is defined to determine the extent of the locality around the sample *x* and is used for explanation. As illustrated in Figure 17, the input image is displayed on the left-hand side, followed by the segmented image portion of GM, WM, and CSF, representing the LIME model's output.

## 3. Discussion

In a comprehensive longitudinal study by Jokinen et al. (2012), the researchers visually evaluated the severity of brain atrophy through MRI. The study revealed that brain atrophy contributes to cognitive decline in patients with small vessel diseases. Furthermore, the research highlighted that medial temporal lobe atrophy (MTLA) subcortical and cortical atrophy exacerbate the impact of white matter lesions (WML) and lacunae on cognitive decline. These findings were corroborated by another MRI study (Nitkunan et al., 2011) that used automated brain volume assessment. It is imperative to closely monitor brain atrophy in patients with small vessel diseases, as it significantly contributes to cognitive decline. By doing so, healthcare practitioners can take proactive measures to prevent further deterioration in cognitive function and improve patient outcomes.

Our work has made significant progress in the field of brain atrophy assessment. We have developed and tested a new pipeline that differs from the end-to-end approach and focuses on linear measurement. A fully automated brain atrophy classification model is developed, incorporating machine learning and deep learning techniques. The classification model can be effectively used for diabetic and brain disorder patients. Clinical information, age, gender, and diabetic patient history were also incorporated into the model, and as a result, the classification performance has improved significantly. This model can be integrated into clinical decision support tools, providing valuable assistance to medical professionals. The motivation for this study is brain atrophy becoming a significant risk factor for brain health with CSVD among prolonged diabetic patients (Mayer et al., 2021). Recent studies (Ter Telgte et al., 2018) have demonstrated that a high strain of white matter hyperintensity (WMH) can significantly impair the integrity of the white matter, ultimately leading to a loss of volume and neurons.

Furthermore, cortical atrophy has been attributed to the neurodegenerative processes triggered by the degeneration of white matter tracts, which can disrupt the functional connections within the brain. These findings emphasize the importance of maintaining optimal brain health and proactively preventing the onset of symptoms related to cerebral small vessel disease. By preventative measures and seeking an early diagnosis, the patient's health and cognitive abilities can be maintained and ensure a higher quality of life.

Our research aims to achieve an essential and innovative goal, which is to implement and validate the approach for automated evaluation of brain atrophy in T2DM on MRI scans of people with type 2 diabetes mellitus. Due to this primary objective, we did not include other diabetes variations or neuroimaging characteristics of cerebrovascular disease patients in our research. This study is the first approach to automate the diagnosis of brain atrophy on MRI images. Using WM, GM, and VS features is an effective method for evaluating brain structure. These features indirectly reflect the structural information of different brain regions, which is crucial for accurately diagnosing brain shrinkage in type 2 diabetic mellitus patients. In the future, the survival rate of the patients can be predicted through statistical and machine learning models by using the features of segmented regions and patient categorical data.

Our work has made significant progress in the field of brain atrophy assessment. We have developed and tested a new pipeline that differs from the end-to-end approach and focuses on linear measurement. Through this pipeline, we have developed a fully automated brain atrophy classification model incorporating machine learning and deep learning techniques. This classification model can be effectively used for diabetic and brain disorder patients. Clinical information, age, gender, and diabetic patient history were also incorporated into the model, and as a result, the classification performance has improved significantly. This model can be integrated into clinical decision support tools, providing valuable assistance to medical professionals. Our motivation for this study was fueled by brain atrophy becoming a significant risk factor for brain health with CSVD among prolonged diabetic patients (Mayer et al., 2021). Recent studies (Ter Telgte et al., 2018) have demonstrated that a high burden of white matter hyperintensity (WMH) can significantly impair the integrity of the white matter, ultimately leading to a loss of volume and neurons.

## Data availability statement

The raw data supporting the conclusions of this article will be made available by the authors, without undue reservation.

## Ethics statement

Ethical approval was not required for the study involving humans in accordance with the local legislation and institutional requirements. Written informed consent to participate in this study was not required from the participants or the participants' legal guardians/next of kin in accordance with the national legislation and the institutional requirements. Written informed consent was not obtained from the individual(s) for the publication of any potentially identifiable images or data included in this article because written informed consent for participation was not required for this study in accordance with the national legislation and the institutional requirements.

## Author contributions

SS: Writing—original draft, Writing—review & editing. SM: Supervision, Writing—review & editing.
